# Barriers and facilitators for implementing peripherally inserted central catheter (PICC) appropriateness guidelines: A longitudinal survey study from 34 Michigan hospitals

**DOI:** 10.1371/journal.pone.0277302

**Published:** 2022-11-04

**Authors:** Gillian Ray-Barruel, Jennifer Horowitz, Elizabeth McLaughlin, Scott Flanders, Vineet Chopra

**Affiliations:** 1 School of Nursing, Midwifery and Social Work, The University of Queensland, St Lucia, Queensland, Australia; 2 Herston Infectious Diseases Institute, The University of Queensland, Herston, Queensland, Australia; 3 Alliance for Vascular Access Teaching and Research, School of Nursing and Midwifery, Griffith University, Nathan, Queensland, Australia; 4 Division of Hospital Medicine, Department of Internal Medicine, Michigan Medicine, Ann Arbor, Michigan, United States of America; 5 The Michigan Hospital Medicine Safety Consortium, Ann Arbor, Michigan, United States of America; 6 Department of Medicine, University of Colorado Anschutz Medical Campus, Aurora, Colorado, United States of America; University of Verona, ITALY

## Abstract

Peripherally inserted central catheters (PICCs) are prevalent devices for medium-to-long-term intravenous therapy but are often associated with morbid and potentially lethal complications. This multi-center study sought to identify barriers and facilitators of implementing evidence-based appropriateness criteria to improve PICC safety and patient outcomes in a pay-for-performance model. Participating hospitals received an online toolkit with five recommendations: establishing a vascular access committee; implementing a clinical decision tool for PICC appropriateness; avoiding short-term PICC use (≤5 days); increasing use of single-lumen PICCs; and avoiding PICC placement in patients with chronic kidney disease. Longitudinal online surveys conducted biannually October 2014–November 2018 tracked implementation efforts. A total of 306 unique surveys from 34 hospitals were completed. The proportion of hospitals with a dedicated committee overseeing PICC appropriateness increased from 53% to 97%. Overall, 94% of hospitals implemented an initiative to reduce short-term and multi-lumen PICC use, and 91% integrated kidney function into PICC placement decisions. Barriers to implementation included: achieving agreement from diverse disciplines, competing hospital priorities, and delays in modifying electronic systems to enable appropriate PICC ordering. Provision of quarterly benchmarking reports, a decision algorithm, access to an online toolkit, and presence of local champion support were cited as crucial in improving practice. Structured quality improvement efforts including a multidisciplinary vascular access committee, clear targets, local champions, and support from an online education toolkit have led to sustained PICC appropriateness and improved patient safety.

## Introduction

Peripherally inserted central catheters (PICCs) enable delivery of intravenous (IV) therapy while providing safe, reliable access for blood draws. Despite their benefits, PICCs are associated with complications, including thromboembolism and bloodstream infection [1–8]. Complications delay treatment and increase length of stay and financial burden for healthcare systems [9].

Despite these risks, inappropriate PICC use, defined as placement in situations where risk outweighs benefits, regardless of cost [10], is common [11–13]. Examples include patients requiring short-term IV therapy [14, 15], patients with prior catheter-related thrombosis [6], and those with kidney dysfunction [16, 17]. Alternative, less invasive devices (e.g., peripheral intravenous or midline catheters) may help avoid inappropriate PICC use and complications [10]. Published in 2015, the Michigan Appropriateness Guide for Intravenous Catheters (MAGIC) provides evidence-based recommendations for use of venous access devices, including PICCs, based on patient-specific factors/clinical scenarios. Adoption of MAGIC in US hospitals has facilitated benchmarking of practice and improved patient safety [18–21].

This study is part of a multi-site project aimed at improving PICC appropriateness and patient outcomes. In 2014, select Michigan hospitals voluntarily joined a collaborative quality improvement (QI) initiative (Michigan Hospital Medicine Safety [HMS] Consortium) to improve PICC use and outcomes. By undertaking routine benchmarking and feedback of data to support improvement, HMS has achieved significant reductions in PICC complications while improving catheter appropriateness [18]. Supported by Blue Cross Blue Shield of Michigan (BCBSM), HMS uses a pay-for-performance model, in which hospitals receive financial incentives to stimulate healthcare improvements in efficiency and quality [22]. In 2014, HMS launched an evidence-based initiative to improve appropriate PICC use in hospitalized patients, emphasizing five recommendations: (1) creating a vascular access committee to review PICC use and outcomes; (2) implementing a clinical decision tool, such as MAGIC [10] or Infusion Nurses Society (INS) Standards of Practice [23], to determine PICC appropriateness prior to insertion; (3) avoiding PICC placement in patients needing venous access for ≤ 5 days; (4) increasing use of single-lumen PICCs/discouraging multi-lumen PICCs; and (5) avoiding PICC placement in patients with estimated glomerular filtration rate (eGFR) < 45 ml/min without nephrology approval.

Hospitals retained flexibility in choosing the intervention approach, sequence, and timing. A toolkit containing resources such as educational webinars, screensavers, badge cards, an online phone App, and ongoing support and follow-up (site visits, teleconferences, etc.) was released in February 2017 [24]. Hospitals were provided with detailed reports showing their site-specific performance compared to other HMS hospitals. These reports highlighted cases not meeting target metrics, and asked hospitals to evaluate these ‘fall-outs’. Finally, quarterly meetings provided progress for the whole collaborative on each recommendation, evidence-based updates, and core content for improvement work.

This study aimed to understand how hospitals engaged in implementation work and identify barriers and facilitators for the successful uptake of PICC appropriateness recommendations.

## Materials and methods

Longitudinal online surveys were conducted biannually between October 2014 and November 2018 to document progress and problems in implementing PICC improvement strategies. As the purpose of HMS is to measure and improve quality of existing practice, this project received a ‘not-regulated’ status from the institutional review board at the University of Michigan (HUM 000163445).

The questionnaires used in this study were developed by the HMS coordinating center and were shared with key stakeholders in HMS that include nursing partners, C suite partners, radiologists, and medical subspecialists. Surveys were administered on-line using Qualtrics®. The HMS program manager emailed a survey invitation to each hospital’s project lead, who was responsible for recording site responses. The project lead was instructed to collect data, as well as barriers and facilitators, from various sources including interviews and focus groups to obtain the most reliable information (e.g., chief quality officer, vascular access teams, bedside nurses, etc.). Each hospital completed one survey per cycle.

Survey completion was tied to hospital pay-for-performance assessment. To track longitudinal progress and implementation of PICC appropriateness strategies, only hospitals completing all nine surveys between Fall 2014 and Fall 2018 were included in this analysis. Each survey contained quantitative and qualitative items, with an average of 11 multiple-choice and 11 open-ended questions. Multiple-choice questions included information on hospital-specific vascular access committees, policies and processes, and QI activities for appropriate device selection. While some open-ended questions sought clarification on the closed-ended questions, they also explored challenges and facilitators to process changes. Survey questions evolved over time to reflect the concurrent work of HMS (S1 File).

Respondents were requested to upload specific documents related to QI work at designated times during the intervention. In Fall 2014, respondents uploaded their hospital PICC policy. In Fall 2016, respondents provided their hospital plan for decreasing number of PICC lumens. In Fall 2017, respondents supplied their hospital decision tool to determine PICC appropriateness. Thus, survey responses were linked to hospital-level policy changes over time.

### Data analysis

Data related to specific strategies (e.g., creation of vascular access committee) was extracted from the multiple-choice responses and the number of hospitals utilizing each strategy was counted. Open-ended survey responses were reviewed by two researchers experienced in qualitative analysis, with no relationship or interaction with survey respondents, and no assumptions/presuppositions of the findings [25]. Each researcher independently conducted a thematic analysis [25], reading responses line-by-line several times to identify the barriers and facilitators to implementing each recommendation. The researchers met regularly to discuss their findings, which were reviewed and confirmed with the project manager. Exemplar quotes were identified to demonstrate the implementation experience.

Uploaded hospital policies were entered into NVivo (NVivo 12 Pro, QSR International, MA) and evaluated for content related to appropriateness criteria (PICC dwell time, number of lumens, and renal function). For each criterion, a range of search terms was utilized to capture related terminology to ensure all appropriate content was tagged. The reviewer then read each policy to ensure all relevant data had been identified. All coding was completed by one researcher and reviewed and confirmed with the second researcher. This paper follows the Revised Standards for Quality Improvement Reporting Excellence (SQUIRE 2.0) [26].

## Results

**Fig 1:**
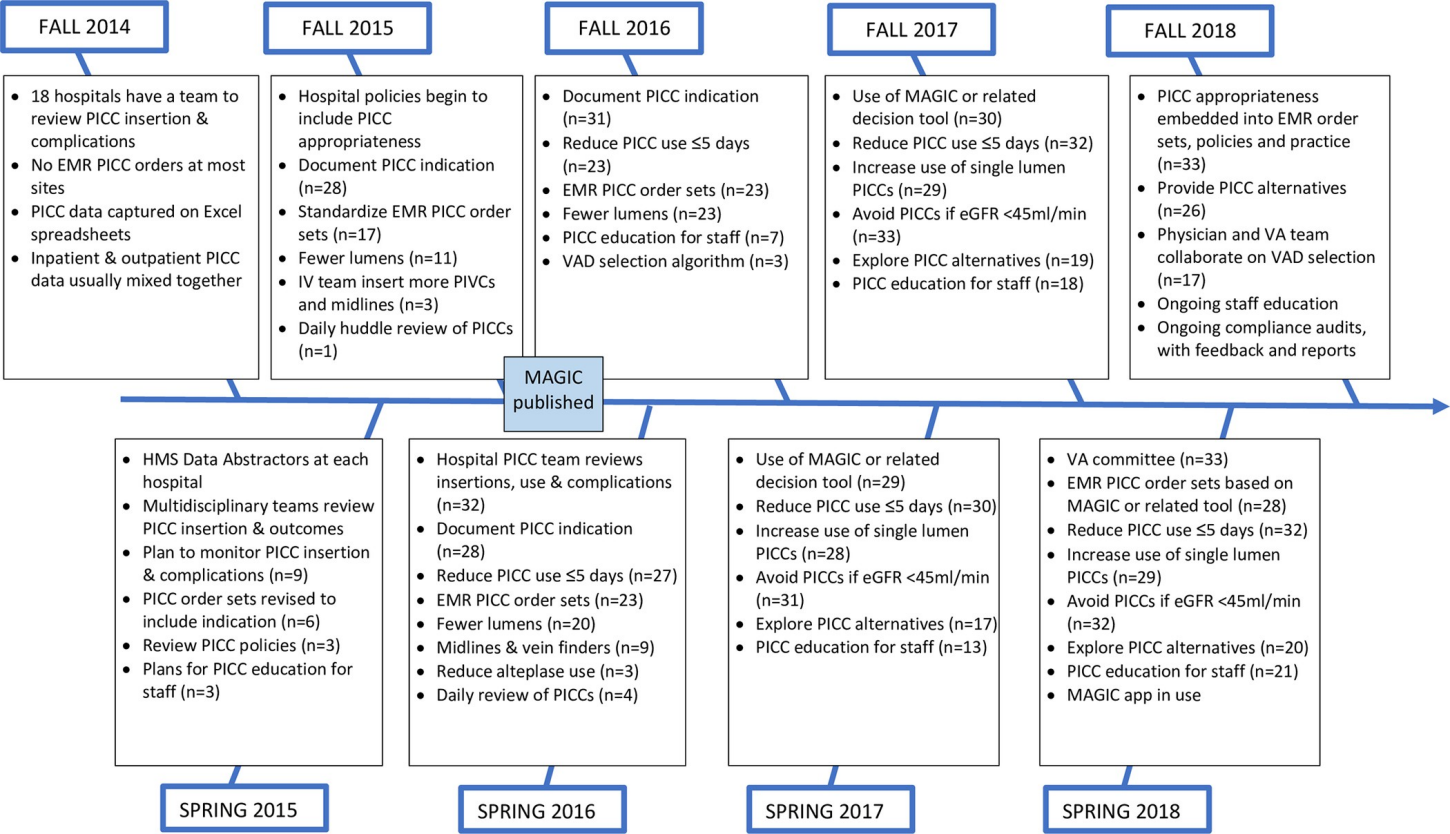
Implementation progress of PICC improvement strategies across 34 Michigan hospitals.

Thirty-seven hospitals were continuously part of HMS between Fall 2014 and Fall 2018; three were excluded because they did not complete all surveys. Thus, 34 hospitals completed all nine surveys, for a total of 306 individual responses. Survey completion ranged between 91% and 100%. Participating hospitals ranged from 49 to 1,085 beds, in various geographic regions of the state (S1 Table). Hospitals employed diverse tactics to meet each recommendation. The implementation progress of PICC improvement strategies is displayed in the fishbone diagram (Fig 1).

### Strategy 1: Convene a vascular access committee to review PICC use and outcomes

In Fall 2014, prior to the release of the MAGIC guidelines, only 18 (53%) hospitals had a committee or team to review PICC outcomes (predominantly infection and thrombosis); no hospitals reviewed PICC appropriateness or had a policy to support PICC decision-making. By 2015, preparatory work had begun, and 26 (76%) hospitals had formed multidisciplinary vascular access committees; 6 (23%) committees met monthly and 15 (58%) met quarterly. By Spring 2017, 33 (97%) hospitals had established a vascular access committee; 12 (36%) met monthly and 19 (58%) met quarterly (Table 1). These 33 hospitals (97%) continued to hold regular meetings in Fall 2018.

**Table 1:** Frequency of vascular access committee meetings^a^.

Component	Fall 2015	Spring 2016	Fall 2016	Spring 2017
	(N = 26)	(N = 32)	(N = 32)	(N = 33)
Bi-Weekly	1 (3.9%)	1 (3.1%)	1 (3.1%)	0 (0.0%)
Monthly	6 (23.1%)	13 (40.6%)	10 (31.3%)	12 (36.4%)
Quarterly	15 (57.7%)	17 (53.1%)	19 (59.4%)	19 (57.6%)
Not Reported	0 (0.0%)	1 (3.1%)	2 (6.3%)	2 (6.1%)

^a^ Question was only asked in four of the surveys

### Reported barriers

Obtaining support from hospital executives, existing teams, and staff was critical to establishing a vascular access committee, but this proved challenging, particularly as hospitals needed data to support the case for a dedicated committee. When the initiative commenced, data available was not granular, and included a composite of “inpatient and outpatient data” (Site 1 & Site 2). Furthermore, PICC outcome data was not routinely collected, making it difficult to evaluate the extent of PICC-related complications. Much early work focused on “determining who needs to be involved based on the data obtained” (Site 2) and “encouraging more physicians to participate in the PICC project and share the data and identify the possibilities for improvement” (Site 3).

### Reported facilitators

Success was facilitated by identifying local champions to drive improvement efforts and ensure stakeholders from various departments were represented. Committee membership included vascular access nurses, interventional radiologists, critical care physicians and nurses, infection control professionals, nephrologists, hematologists/oncologists, epidemiologists, quality and safety professionals, VTE experts and pharmacists.

Participation in a hospital collaborative enabled benchmarking and targeted performance improvement; sites used “HMS data to build a business case for Vascular Access Team” (Site 4). With HMS data collection tools and support, local committees began to monitor PICC insertion, use, and complications. In return, HMS provided quarterly PICC performance reports, enabling hospitals to track progress, identify areas for improvement, and benchmark complication rates with other sites. Hospitals distributed HMS reports at committee meetings, vascular access team rounds, staff huddles, and in newsletters. These data were reportedly crucial to improving hospital practice; hospitals described increasing QI PICC activities and began linking their activity to ongoing efforts to reduce infection. For example, daily review of patients with a PICC by infection control or vascular access nurses and regular PICC audits by ‘line champions’ emerged, with data shared across teams via dashboards: “Within the past 6 months, the daily huddle has begun to review PICC lines. They review PICC indications, the necessity of continuation, any complications, and physicians are called to discuss concerns or alternate [sic] venous access methods” (Site 5).

### Strategy 2: Use MAGIC or a related decision-tool to determine PICC appropriateness prior to device placement

In Fall 2014, although 33 (97%) hospitals had PICC policies and/or procedures, PICC appropriateness was sparsely addressed: e.g., dwell time (n = 10, 37%), number of lumens (n = 8, 30%), and kidney function (n = 3, 11.1%). Following publication of MAGIC^7^ in late 2015, hospitals began to adopt PICC appropriateness criteria. This required revision of PICC policies, incorporation of PICC orders in electronic medical records (EMRs), and staff education.

### Reported barriers

Previously, PICC insertion notes were “handwritten and scanned into the EMR. Management notes are EMR” (Site 6) and “documentation was scattered, missing and inconsistent, as the PICC team did not have access to the standard PICC insertion note in the EMR” (Site 7). Once quarterly HMS reports became available, the shortfalls in PICC policies and documentation became evident: “Our data showed approx. 60% of PICCs placed were for an unknown reason. We needed to improve the documentation process before we could further evaluate the appropriateness of placement. The main cause of this problem was that physicians ordering device were not documenting reason for insertion” (Site 7).

Incorporating PICC guidelines into EMRs was challenging, as hospitals experienced delays in securing information technology (IT) support for EMR adaptations: “the speed of the required IT changes regarding PICC ordering and selection of the appropriate access seems to be our most difficult obstacle” (Site 8). Competing priorities for EMR updates were another delay: “We have attempted to change the order set to include the risk score, but due to changing of [electronic medication administration record] within the next few months, it must also be agreed to by all hospitals within the corporation. It has been denied” (Site 9).

Much effort was expended on getting agreement from physicians and radiology to enact change. Initially, some providers were reluctant to implement MAGIC guidelines: “[This] hospital did not have a difficult access related decision tool to determine PICC appropriateness, and providers would not buy into using the MAGIC tool” (Site 7). A further challenge was identified in achieving hospital-wide compliance with the algorithm: “Another obstacle is the buy-in to use the algorithm when deciding on appropriate selection of access especially upon discharge from ICU” (Site 8). Implementing change with staff turnover and new hires necessitated ongoing education: “extensive education on PICC care and maintenance” (Site 4).

### Reported facilitators

By Spring 2015, hospitals had begun to review PICC practices and update local policies, including EMR ordering and documentation: “All of the facilities are working collectively with IT to improve PICC order sets or create them, and add in a drop-down for PICC indications” (Site 10). By mid-2018, a PICC order set had been integrated into EMRs at many sites, with hospital-wide education and follow-up to ensure compliance: “We initiated a PICC order set at the end of June 2018 that mirrors the MAGIC guidelines, and we are working with the residents, nurses and attendings to make sure everyone is compliant using the order set. Letters are sent to those who are not compliant and if they are a resident a copy is sent to their attending” (Site 8).

To avoid inappropriate PICC insertion, hospital policies were updated to encourage consideration of alternative vascular access devices, such as midline catheters; by Spring 2017, 16 (47%) hospitals reported building or enhancing their midline program. By Fall 2018, 23 (68%) hospitals were routinely placing midlines: “a midline policy in place is based on the MAGIC tool. The vascular access team also assesses every patient for line appropriateness prior to insertion of a PICC” (Site 11). In addition to investing in different devices, more autonomy in decision-making was emerging for frontline vascular access nurses: “Using MAGIC as a guideline for placing PICC lines, our committee mandated final decision-making for line placement to fall with the vascular access nurse who is the last person to evaluate the patient needs and safety before the line is placed” (Site 6).

In February 2017, HMS published a PICC toolkit and online education resources that hospitals could share with staff; by the following Fall, 30 (88%) hospitals reported using appropriateness criteria to guide device choice before insertion. A pre-insertion pause to ensure appropriate device selection had become accepted practice at 23 (68%) hospitals. One hospital used MAGIC to create a decision algorithm for PICC appropriateness,^12^ which was shared among other sites who reported “laminating and hanging the algorithm in the appropriate areas as a reference” (Site 8).

By Spring 2018, almost all vascular access teams used the MAGIC smartphone application (free download) and educated nursing and medical staff on appropriate device selection via education sessions, medical grand rounds, and staff huddles, with one hospital reporting “resident/physician education required yearly regarding PICC line appropriateness” (Site 8). Computer screensavers emphasized the importance of PICC appropriateness. In addition to “ongoing education of staff regarding guidelines, protocols, best practice, and data collection” (Site 12), hospitals were also making “changes to the way we educate our patients on PICCs” (Site 13), in accordance with appropriateness criteria. Fall-outs continued to be monitored monthly by the HMS abstractor at each site in conjunction with the local vascular access committee, with regular feedback and reports seen as beneficial “to identify areas to focus on for quality improvement” (Site 14).

### Strategy 3: Improve appropriate PICC use

As committee monitoring of vascular access practices expanded, all hospitals implemented activities targeting reduction in short-term and multi-lumen PICC use and avoidance of PICCs in patients with chronic kidney disease. PICC policies and EMR order sets were updated to include prompts for appropriate indication, expected duration, defaults for single-lumen PICCs, and nephrology consultation for patients with eGFR <45 ml/min. The following examples illustrate reported barriers and facilitators encountered while implementing process changes to improve appropriate PICC use.

#### a. Reduce PICC use in patients requiring vascular access for five days or less

Hospitals leveraged diverse strategies to achieve this objective; by Spring 2018, 32 (94%) hospitals reported targeting short-term PICC use (≤5 days).

*Reported barriers*. Despite overall positive comments, achieving compliance from staff posed an ongoing challenge for some. For example, “Critical care is resistant to use an alternate [sic] central venous access. State PICC is less expensive and easier to place. No physician is needed for placement. And usually no radiology is required. Also, vascular access team is uncertain why PICC lines placed <5 days is a problem. Their responses are as follows: causes less trauma to patient than a triple-lumen central line; more staff are available to place PICC lines; less expertise is needed for PICC lines” (Site 6).

*Reported facilitators*. Moving hospitals away from PICCs as a short-term vascular access solution involved a cultural change in thinking about appropriate device choice prior to placement. Device appropriateness training was provided for nurses and physicians, the hospital vascular access policies were updated, and a PICC decision algorithm was implemented: “We incorporated decision-support tools in the new PICC placement order set that require documentation by the provider that states the reason(s) for short-term use” (Site 15). Short-term PICC use was monitored and discussed at team meetings, with further education as needed.

Another strategy was evaluation of vascular access device use in patients with difficult venous access. Prior to the project, these patients routinely had a PICC placed at all participating sites. As hospitals evaluated the complication data provided by HMS, they shifted their focus by improving staff competency around placement of peripheral intravenous catheters (PIVCs) using infra-red vein finders and ultrasound technology, and moving towards clinical rather than routine PIVC replacement: “Difficult access seems to be the most common indication for PICC use <5 days. We have worked with educating nursing staff and physicians. The hospital purchased vein finders and taught staff how to use. We completed training on ultrasound-guided IV starts with our rapid response nurses, who are the go-to in difficult starts” (Site 16).

Some sites increased vascular access team capacity and trained nurses to insert midline catheters as a PICC alternative; as midline insertions increased, hospitals witnessed a corresponding decrease in short-term PICCs: “We now have longer peripheral IV catheters available […] a 10-day catheter may be able to take the place of a PICC in certain situations” (Site 17), and “[Hospital] has given inserting providers the ability to determine the most appropriate vascular access device for each patient. This has led to increased midline use overall while decreasing short-term PICC use” (Site 10).

Teams also reviewed PICC use for antibiotic therapy. By examining their HMS data, they identified many IV antibiotics were unwarranted or could have been administered orally, and policies and orders emerged to mandate infectious diseases consultation prior to PICC insertion. Additionally, by 2017, five hospitals reported the decision to place a device had evolved from physician-only to a joint decision between vascular access nurses and the attending physician: “The vascular access nurse and ordering physician discuss the indication for the PICC and determine if it is appropriate and if the dwell time will be >5 days” (Site 13).

#### b. Increase use of single-lumen PICCs and discourage multi-lumen PICCs

Between Spring 2017 (27/34; 79%) and Fall 2018 (32/34; 94%), a growing number of hospitals adopted initiatives promoting use of single-lumen PICCs and discouraging multi-lumen PICCs.

*Reported barriers*. Changes to hospital policies and order-sets alone were not enough to overcome the culture where multi-lumen PICCs were inserted regularly to provide a “back up lumen.” Some staff were resistant to using single-lumen PICCs, particularly for patients requiring regular blood draws: “There is a lack of communication between providers, and at times the providers are resistant to the vascular access team’s recommendation of another type of access and/or the number of lumens. Double-lumen PICCs are requested for obtaining blood for lab draws when the patient has poor access and is on an IV drip” (Site 18).

*Reported facilitators*. Eleven sites reported that vascular access teams had implemented a lumen criteria decision process and, in addition to making the final decision regarding device choice, the team assumed responsibility for choosing and monitoring the number of PICC lumens: “The vascular access team does a chart review and assesses the patient prior to selecting the number of lumens placed, including medications the patient has/plan to receive. A lumen criteria information sheet has been developed that is in alignment with INS and MAGIC standards. Education for providers is being developed which addresses the complications of multi-lumen PICCs and alternative vascular access devices to consider” (Site 18).

Others leveraged the EMR, changing order sets to default to single-lumen PICCs and requiring justification (and auditing) of exceptions. To enhance single-lumen use, three hospitals added pharmacists to their vascular access committee and started to coordinate infusion orders with device selection; two hospitals reported mandating pharmacy review of orders for multi-lumen PICCs. Innovations that emerged from engaging pharmacists included spreading out incompatible medications while using a single-lumen PICC or increasing dilution of medications for safe peripheral administration: “Our PICC placement order set includes default selection of single-lumen PICC. We partnered with inpatient pharmacy and our vascular access nurses to create a list of appropriate indications for multi-lumen PICCs. We piloted a process that involves pharmacy review of requests for multi-lumen PICCs to verify appropriate use” (Site 15). Sites also revised their flushing policy and monitored alteplase usage.

#### c. Avoid PICC placement in patients with eGFR <45 ml/min, where possible

In Fall 2014, only 3 (11.1%) hospitals had included kidney function in their PICC policy. By Spring 2017, 31 (91%) hospitals reported integrating kidney function into PICC placement decisions, including a requirement for nephrology consultation for patients with eGFR <45 ml/min.

*Reported barriers*. There was a widespread lack of understanding that renal function needed consideration prior to PICC placement in patients with chronic kidney disease: “Providers have questioned the evidence behind using eGFR less than 45 and how that number was determined. Providers question why nephrology clearance is required and not vascular surgery clearance. Providers question what matters most–acutely treating patients and not delaying necessary lines while waiting for nephrology clearance. The questions and concerns expressed continue to prompt much discussion on these topics” (Site 5).

A reported barrier was obtaining buy-in from nephrologists, who would need to become more engaged with PICC selections: “Our nephrology services are provided by a consulting group and it has been difficult to get active involvement from this group regarding policy/guideline development” (Site 12). Once nephrology support was obtained, lag in hospital policy updates, communication challenges, and competing priorities among teams were cited as barriers to implementation of PICC appropriateness for this patient cohort: “At this time [Spring 2017], there is no policy in place that states the PICC team needs nephrology approval for eGFR <45. In the ICU, physicians may prefer to order PICC lines without the delay of consulting nephrology. There is a lack of communication between the services. The HMS eGFR measure has brought this topic to the forefront and prompted discussion and reconsideration of the current practices” (Site 5). At smaller hospitals, a lack of nephrologists was an obstacle: “Since we are a small hospital, there is generally one nephrologist who makes rounds–adding consults for nephrology approval for PICC placement adds to their workload” (Site 18).

*Reported facilitators*. Hospitals revised PICC policies to support nephrology consultation prior to PICC placement in relevant patients, utilized local champions, and implemented collaborative educational documents to share with ordering providers and inserters: “Our PICC RNs [registered nurses] check lab results before placing PICCs and if the eGFR is <45 they contact necessary providers to determine appropriateness … Our physician champion has educated all residents and hospitalists to order a nephrology consult for all patients with an eGFR less than 45 ml/min when considering PICC placement. Additionally, our PICC team reviews all PICC cases placed in patients with an eGFR <45, and our physician champion provides direct feedback to individual providers with fall-outs” (Site 7).

Hospitals implemented EMR PICC order set modifications to heighten awareness of eGFR prior to PICC insertion, including auto-filling eGFR when a PICC order is activated, flagging patients with eGFR that is a contraindication to PICC placement, or alerting nephrology when PICC consult is needed: “Our order sets ‘fire an alert’ if the eGFR is less than 45ml/min” (Site 17).

## Discussion

Between 2014 and 2018, we conducted biannual surveys in 34 Michigan hospitals to identify how sites implemented PICC safety and appropriateness initiatives. By 2018, 33 of 34 hospitals had vascular access committees and all had adopted PICC improvement strategies. Two-thirds reported implementing MAGIC or a related decision-tool for PICC appropriateness. Work to reduce short-term and multi-lumen PICC use and PICC placement in patients with chronic kidney disease evolved, with hospitals innovating using evidence, clinical context, and benchmarked data to spur performance. Ongoing measurement has demonstrated sustained patient safety benefits, which we have published previously [18, 19, 27], showing that, with the appropriate strategy, evidence can translate into clinical practice.

Implementing and sustaining change requires time, effort, and resources. The survey respondents identified an array of strategies undertaken to implement PICC appropriateness, including training vascular access teams in midline catheter placement and the use of ultrasound and near-infrared technologies. A crucial challenge identified by respondents was obtaining consensus from diverse stakeholders. Engaging stakeholders and obtaining their ongoing commitment is important for any implementation project because local experts can identify the potential barriers and effects of process change [28]. This was achieved by designating local champions (frontline workers), establishing multidisciplinary vascular access committees, providing PICC usage and outcomes data, updating hospital policies, and creating or modifying EMR PICC order sets. The latter also proved challenging and time-consuming. Electronic order sets for central line insertion improve staff compliance with clinical guidelines, with significant improvements in documentation [29], as well as reductions in central-line bloodstream infections [30–32] and unnecessary PICC placement [33]. Recommended processes for streamlining EMR updates include: achieving senior leadership buy-in with the use of outcome data to emphasize organizational need for updates [34]; seeking input and feedback on proposed EMR data fields from interdisciplinary clinical experts as well as information technology throughout the change cycle [34]; bundling requested changes to reduce downtime for system updates; and providing staff training on EMR changes and optimum use of order sets [34].

Introduced as a lower risk alternative to non-tunneled central venous access devices, PICCs may lead to venous thrombosis, occlusion, and bloodstream infection [1–8]. Prior to the implementation of MAGIC in late 2015, variation in PICC practices was common and discordant with clinical guidelines [19, 35–37]. Up to 24% of PICCs were inserted for short-term use (≤ 5 days) in patients with difficult venous access, and multi-lumen PICCs were inserted when only one lumen was needed [37]. Alarmingly, 19% of patients sustained a PICC-related complication [37]. We recognize that no vascular access device is risk-free, and PICCs are an appropriate vascular access choice for many patients who require reliable venous access; however, the associated risks should be understood and taken into consideration when selecting the appropriate device for each patient. A recent meta-analysis identified that PICCs inserted with current best practices (smaller diameter, fewer lumens, dedicated insertion teams, evidence-based insertion and maintenance bundles) have lower thrombosis and infection risks than centrally inserted central catheters [38].

Our study shows how QI efforts—such as engagement of key stakeholders; refinement of processes/policies; creation and updating EMR order sets; provision of education; and collaboration with end-users—can successfully improve PICC use and outcomes across a diverse range of hospitals. Overall PICC complications decreased from 14.7% in 2013 to 10.7% in 2020 [18]. Introducing clear goals and providing support to sites, without mandating a ‘one-size-fits-all’ approach, offered flexibility for hospitals to adopt strategies as able. This structure also afforded a scaffolding on which nursing constituents (who are often marginalized in decision-making) could take a more central role in crafting policy and practice recommendations. Consistent with this, many hospitals began to empower vascular access teams for key decisions including number of lumens, device choices, and when to consult specialists before device insertion. Vascular access teams have repeatedly demonstrated value in making decisions about PICC use and providing nursing education for PICC and midline catheter management by bedside registered nurses [39–41], as well as developing unit-based vascular access champions and providing training in PIVC insertion [42].

A recent editorial called for scaling up initiatives such as MAGIC to other healthcare organizations, both in the US and internationally [43]. Our work has implications for hospitals planning to implement similar initiatives. First, our findings show the importance of a data-driven approach to improving clinical processes, quality, and safety. Second, establishing local governance via a central committee to engage key stakeholders to examine data and identify gaps proved invaluable. Thirdly, understanding challenges and facilitators for adoption was important. By remaining flexible and supporting hospitals with an online toolkit, education, and data resources, each site was empowered to tackle implementation in a tailored fashion. And finally, local champion support was essential. This paper reports how we achieved practice change across many facilities. We hope the insights discussed here will help others engaging in large scale QI work benefit from the organization, structure, data-driven and collaborative approach we have developed in Michigan.

### Study strengths and limitations

This is the first study to evaluate how a consortium of hospitals implemented evidence-based strategies to improve PICC safety and patient outcomes. A particular strength is the longitudinal survey design, capturing implementation processes of a large-scale quality program across multiple hospitals over several years. However, our study has limitations. The model of a group of hospitals with pay-for-performance methods possibly limits generalizability. Yet, more healthcare systems are integrating in the US and value-based initiatives are highly prevalent; our report provides a blueprint for implementing large-scale, multi-site QI initiatives under these constructs. Also, our approach used surveys with drop-down lists and free-text responses to identify barriers and facilitators, which may not have captured the full range of challenges experienced. However, as consistent themes (getting buy-in, adapting EMR, etc.) were identified by many respondents in a variety of settings over several years, we are confident that responses captured the main barriers.

## Conclusions

Multidisciplinary vascular access committees have become standard practice, and over two-thirds of US hospitals have implemented MAGIC or a related decision tool, resulting in improved PICC stewardship and appropriateness. The challenges and facilitators reported here will inform implementation efforts for other hospitals planning to improve outcomes for patients who need safe, reliable vascular access.

## Supporting information

S1 File. Survey questions (PDF)

S1 Table Hospital demographics. (DOCX)
