# Supplementary material for: Barriers and facilitators for implementing peripherally inserted central catheter (PICC) appropriateness guidelines: A longitudinal survey study from 34 Michigan hospitals
Source: PLoS One. 2022 Nov 4;17(11):e0277302. doi: 10.1371/journal.pone.0277302 (PMC9635738; doi:10.1371/journal.pone.0277302)
Supplement: S1 File. Survey questions — (PDF) [file pone.0277302.s001.pdf]

Survey Questions

Barriers and facilitators for implementing peripherally inserted central catheter (PICC) appropriateness guidelines: A longitudinal survey study from 34 Michigan hospitals

Gillian Ray-Barruel, RN PhD, Jennifer Horowitz, MA, Elizabeth McLaughlin, RN, MS, Scott Flanders, MD, Vineet Chopra, MD, MSc

Additional file 1 Table of Contents

	Page
HMS Tier 1 Qualitative Survey Topic Questions 2014–2018	2
HMS Quality Improvement Survey Questions	3
Fall – 2014	3
Spring – 2015	5
Fall – 2015	6
Spring – 2016	8
Fall – 2016	11
Spring – 2017	13
Fall – 2017	17
Spring – 2018	21
Fall – 2018	25

HMS Tier 1 Qualitative Survey Topic Questions 2014–2018

**Questions asked**	**Fall 2014**	**Spring 2015**	**Fall 2015**	**Spring 2016**	**Fall 2016**	**Spring 2017**	**Fall 2017**	**Spring 2018**	**Fall 2018**
QI Activity (PICC specific)	x	x	x	x	x				
Anything else you would like the Coordinating Center to know about PICC insertions at your hospital?	x								
Other QI activities (PICC specific)		x	x	x	x	x	x	x	x
Plan to utilize HMS PICC data		x	x	x					
Flush frequency			x	x					
Flush other			x	x					
Plan to decrease number of lumens and gauge size of PICCs					x				
Anything else you would like the HMS Coordinating Center to know about the activities at your hospital?					x	x	x		
QI Activity (PICC Tier 1 specific)						x	x	x	x
QI Activity related to MAGIC or INS Standards for PICC						x	x	x	x
QI project to reduce short-term PICC use						x	x	x	x
QI project to reduce multi-lumen PICCs						x	x	x	x
QI project to avoid PICC if eGFR <45ml/min						x	x	x	x
Barriers encountered r/t PICC Interventions						x	x	x	x
Feedback/suggested improvements for future webinars focusing on PICC interventions.						x			
You indicated a need for PICC specific tools/interventions, please describe.								x	x

<u>**HMS Quality Improvement Activity - Fall 2014**</u>

Please answer the following questions based on the activity at your hospital over the last six months. The survey is due November 14, 2014.

1. Hospital Name

PICC Initiative Baseline Hospital Information

2. Will your VTE physician champion also be your PICC physician champion?
   - Yes
   - No
3. If no, have you identified a PICC champion?
   - Yes
   - No
4. If yes, please indicate the physician's name and e-mail address.
5. Does your hospital currently have a multi-disciplinary team that reviews PICC care and quality concerns?
   - Yes
   - No
6. Approximately what percentage of PICCs inserted at your hospital are inserted by the following groups?

	Not Applicable	1-25%	26-50%	51-75%	76-100%
Vascular Access Team	O	O	O	O	O
Interventional Radiology (IR)	O	O	O	O	O
Nurse Practitioners/Physician Assistants	O	O	O	O	O
Physicians (non-IR)	O	O	O	O	O
Other	O	O	O	O	O

8. What was the total number of PICCs placed in adult inpatients at your hospital over the last 12months? (Please use data from the most current 12 months available.)

9. Does your hospital have an electronic medical record that includes PICC insertion notes and/or PICC management notes?

   - Yes
   - No

10. Does your hospital have any PICC guidelines/policies/procedures?

   - Yes
   - No

11. If yes, please upload the PICC guidelines/policies/procedures.

12. Will you be required to submit the PICC initiative to your institutional review board (IRB) (even though it is quality improvement and not research)?

   - Yes
   - No

13. Anything else you would like the Coordinating Center to know about PICC insertions at your hospital?

<u>HMS Quality Improvement Activity - Spring 2015</u>

Please answer the following questions based on the activity at your hospital over the last six months.

The survey is due June 5, 2015.

1. Hospital Name

PICC Specific Information

2. How many physicians at your institution are directly involved with the HMS PICC Project that are not already part of the VTE prophylaxis Project?
    - Yes
    - No
3. Does your hospital currently have a multi-disciplinary team that reviews PICC care and quality concerns?
    - Yes
    - No
4. If your hospital currently has a multi-disciplinary team that reviews PICC care and quality concerns, please describe the quality improvement work in more detail.
5. How often does the multi-disciplinary team meet to review PICC care and quality concerns?
    - Quarterly
    - Monthly
    - Bi- Weekly
    - None of the above
6. Has your hospital developed a plan for how to utilize the PICC data provided by HMS?
    - Yes
    - No
7. If your hospital has developed a plan for how to utilize the PICC data provided by HMS, please describe this plan in more detail.
8. Does your hospital have any PICC guidelines/policies/procedures?
    - Yes
    - No
9. If yes, please upload the PICC guidelines/policies/procedures.

<u>HMS Quality Improvement Activity - Fall 2015</u>

Please answer the following questions based on the activity at your hospital over the last six months (unless otherwise specified). The survey is due November 4, 2015.

1. Hospital Name

PICC Specific Information

2. How many physicians at your institution are directly involved with the **HMS PICC Initiative** that are not already part of the VTE prophylaxis Project?

3. What was the total number of PICCs placed in adult inpatients at your hospital over the last 12 months? Please use data from the most current 12 months available. Please exclude midlines or catheter exchanges if possible. If not possible, please check the box below indicating that exchanges were included.

4. Click below if relevant

   - Exchanges included in total PICC number above.

5. Please tell us the source of the information related to the total number of PICCs. (Select all that apply.)

   - Vascular Access Team
   - Interventional Radiology Team
   - Billing/Financial Records
   - Material Supplies/Central Sterile Supply
   - Other

6. Has your hospital worked on any of the following quality improvement projects related to PICCs in the last 12 months? (Check all that apply.)

   - Changes to Device Type (number of lumens, gauge)
   - Improvements in Documentation (Indication for PICC, Clinical Reasons)
   - Changes to Electronic Medical Record (For Ordering or Documenting Reasons for PICC Placement)
   - Other (describe below)

7. Please describe your hospital's other quality improvement efforts related to PICCs. (If "other" quality improvement project related to PICCs identified)

8. Does your hospital currently have a multi-disciplinary team that reviews PICC care and quality concerns?

   - Yes
   - No

9. If yes, how often does the multi-disciplinary team meet to review PICC care and quality concerns?

- Quarterly
- Monthly
- Bi- Weekly
- None of the above

10. Has your hospital developed a plan for how to utilize the PICC data provided by HMS?

- Yes
- No

11. If your hospital has developed a plan for how to utilize the PICC data provided by HMS, please describe this plan in more detail.

12. Does your hospital have a nursing flushing protocol for PICCs?

- Yes
- No

13. If yes, please upload the nursing flushing protocol.

14. What does your hospital use for flushing PICCs?

- Normal Saline
- Heparin
- Normal Saline & Heparin
- Other

15. What is the standard for how often you flush PICCs at your hospital?

16. What is the standard for flushing PICCs before/after phlebotomy?

- Yes, our standard is to flush
- No, our standard is to not flush

17. Any other information regarding PICC flushing that you would like to share?

18. Does your hospital have a policy or protocol for transfusing blood through a PICC?

- Yes
- No

19. If yes, please upload the transfusion policy or protocol.

<u>HMS Quality Improvement Activity - Spring 2016</u>

Please answer the following questions based on the activity at your hospital over the last six months (unless otherwise specified). The survey is due July 1, 2016.

1. Hospital Name

PICC Specific Information

2. How many physicians at your institution are directly involved with the HMS PICC Initiative that are not already part of the VTE prophylaxis Project?

3. What was the total number of PICCs placed in adult inpatients at your hospital over the last 12 months? Please use data from the most current 12 months available. Please exclude midlines or catheter exchanges if possible.

4. Indicate the following:

   - No exchanges or midlines included in total PICC number above
   - Exchanges included in total PICC number above.
   - Midlines included in total PICC number above.
   - Exchanges and midlines included in total PICC number above

5. Please tell us the source of the information related to the total number of PICCs. (Select all that apply.)

   - Vascular Access Team
   - Interventional Radiology Team
   - Billing/Financial Records
   - Material Supplies/Central Sterile Supply
   - Other

6. Has your hospital worked on any of the following quality improvement projects related to PICCs in the last 12 months? (Check all that apply.)

   - Changes to Device Type (number of lumens, gauge)
   - Improvements in Documentation (Indication for PICC, Clinical Reasons)
   - Changes to Electronic Medical Record (For Ordering or Documenting Reasons for PICC Placement)
   - Reducing PICCs inserted for 5 days or less
   - Preventing PICC Complications
   - Other (describe below)

7. Please describe your hospitals other quality improvement efforts related to PICCs. (if "other" was selected in "Has your hospital worked on any of the following quality improvement projects related to PICCs in the last 12 months?")

8. For quality improvement work related to preventing PICC complications, which complication? (if "preventing PICC complications" was selected in "Has your hospital worked on any of the following quality improvement projects related to PICCs in the last 12 months?")

   - Pulmonary Embolism (PE)
   - Deep Vein Thrombosis (DVT)
   - Central Line Associated Blood Stream Infection (CLABSI)
   - Catheter Occlusion
   - Other

9. Does your hospital currently have a multi-disciplinary team/committee that reviews PICC care and quality concerns?

   - Yes
   - No

10. For the multi-disciplinary team/committee that reviews PICC care and quality concerns, indicate the name of the team (e.g. CLABSI Committee) (If "yes" to "Does your hospital currently have a multi-disciplinary team/committee that reviews PICC care and quality concerns?")

11. How often does the multi-disciplinary team meet to review PICC care and quality concerns? (If "yes" to "Does your hospital currently have a multi-disciplinary team/committee that reviews PICC care and quality concerns?")

    - Quarterly
    - Monthly
    - Bi- Weekly
    - None of the above

12. Has your hospital developed a plan for how to utilize the PICC data provided by HMS?

    - Yes
    - No

13. If your hospital has developed a plan for how to utilize the PICC data provided by HMS, please describe this plan in more detail.

14. Does your hospital have a flushing protocol for PICCs?

    - Yes
    - No

15. If yes, please upload the PICC flushing protocol (Please upload the PICC flushing protocol not the PICC insertion policy).

16. What does your hospital use for flushing PICCs?

    - Normal Saline
    - Heparin
    - Normal Saline & Heparin
    - Other

17. What volume of flush is typically used for each episode of flushing PICCs?

- 3 ml
- 5 ml
- 10 ml
- Other

18. What is the standard for how often you flush PICCs at your hospital?

19. Does your hospital use pre-filled syringes for flushing PICCs?

- Yes
- No

20. For the pre-filled syringes, indicate the name of the manufacturer: (if "yes" to "Does your hospital use pre-filled syringes for flushing PICCs?")

21. Instead of pre-filled syringes, what does your hospital use? (if "no" to "Does your hospital use pre-filled syringes for flushing PICCs?")

22. What is the standard for flushing PICCs before/after phlebotomy?

- Yes, our standard is to flush
- No, our standard is to not flush

23. Who flushes PICCs after phlebotomy?

- Phlebotomist
- Bedside RN
- Tech/Nurses Aide
- Other

24. Does your hospital use connectors for IV infusions through PICCs?

- Yes
- No

25. For the connectors for IV infusions through PICCs, indicate the type of connector: (if "yes" to "Does your hospital use connectors for IV infusions through PICCs?")

- Neutral Valve
- Positive Displacement
- Negative Displacement
- Other

26. Any other information regarding PICC flushing that you would like to share?

27. Does your hospital have a policy or protocol for transfusing blood through a PICC?

- Yes
- No

28. If yes, please upload the transfusion policy or protocol.

<u>HMS Quality Improvement Activity - Fall 2016</u>

Please answer the following questions based on the activity at your hospital over the last six months (unless otherwise specified). The survey is due November 7, 2016.

1. Hospital Name

PICC Specific Information

2. How many physicians at your institution are directly involved with the HMS PICC Initiative that are not already part of the VTE prophylaxis Project?

3. What was the total number of PICCs placed in adult inpatients at your hospital over the last 12months? Please use data from the most current 12 months available. Please exclude midlines or catheter exchanges if possible.

4. Indicate the following:

    - Exchanges included in total PICC number above.
    - Midlines included in total PICC number above.
    - None of the above

5. Please tell us the source of the information related to the total number of PICCs. (Select all that apply.)

    - Vascular Access Team
    - Interventional Radiology Team
    - Billing/Financial Records
    - Material Supplies/Central Sterile Supply
    - Other

6. Has your hospital worked on any of the following quality improvement projects related to PICCs in the last 6 months? (Check all that apply.)

    - Changes to Device Type (number of lumens, gauge)
    - Improvements in Documentation (Indication for PICC, Clinical Reasons)
    - Changes to Electronic Medical Record (For Ordering or Documenting Reasons for PICC Placement)
    - Reducing PICCs inserted for 5 days or less
    - Reducing PICCs inserted in those with an eGFR less than or equal to 45
    - Preventing PICC Complications
    - Other (describe below)

7. Please describe your hospital's other quality improvement efforts related to PICCs. (If "other" was identified as a quality improvement project related to PICCs in the past six months).

8. For quality improvement work related to preventing PICC complications, which complication? (If "preventing PICC complications" was identified as a quality improvement project related to PICCs in the past six months).

- Pulmonary Embolism (PE)
- Deep Vein Thrombosis (DVT)
- Central Line Associated Blood Stream Infection (CLABSI)
- Catheter Occlusion
- Other

9. Please upload your hospital's plan for decreasing the number of lumens and gauge size of PICCs used.

10. Does your hospital currently have a multi-disciplinary team/committee that reviews PICC care and quality concerns?

- Yes
- No

11. If yes, how often does the multi-disciplinary team meet to review PICC care and quality concerns?

- Quarterly
- Monthly
- Bi- Weekly
- None of the above

12. Has any HMS-produced PICC data been shared with your institution (i.e. reports distributed at the HMS collaborative wide meetings and/or hospital level reports in the HMS database)?

- Yes
- No

HMS Quality Improvement Survey - Spring 2017

As a reminder, the HMS Spring 2017 Quality Improvement Survey contains several questions that pertain to the 2017 Michigan Hospital Medicine Safety Consortium Collaborative Quality Initiative Performance Index Scorecard. Please note, that you will have an additional opportunity to answer these questions on the HMS Fall 2017 Quality Improvement Survey. If you have any questions related to this, please contact the HMS Project Manager, Elizabeth McLaughlin (emcnair@med.umich.edu).

Please answer the following questions based on the activity at your hospital over the last six months (unless otherwise specified). The survey is due July 7, 2017.

1. Hospital Name

PICC Specific Information

2. What was the total number of PICCs placed in adult hospitalized patients by all PICC inserters (e.g. interventional radiology, vascular access, etc.) at your hospital over the last 12 months? Please use data from the most current 12 months available. Please exclude midlines or catheter exchanges, if possible.

3. Indicate the following:

   - Exchanges included in total PICC number above.
   - Midlines included in total PICC number above.
   - None of the above

4. Please tell us the source of the information related to the total number of PICCs. (Select all that apply.)

   - Vascular Access Team
   - Interventional Radiology Team
   - Billing/Financial Records
   - Material Supplies/Central Sterile Supply
   - Other

5. You indicated "Other" as the source of the information related to the total number of PICCs. Please describe the "Other" method.

6. Which of the following quality improvement project(s) related to the PICC Tier 1 Interventions has your hospital been actively working on? (Check all that apply.)

   - Creation of a vascular access committee
   - Use of MAGIC or a related decision-tool (e.g. INS Standards) to determine PICC appropriateness
   - Reducing Short Term PICC Use
   - Increasing use of single lumen PICCs; decreasing use of multi-lumen PICCs
   - Avoiding PICC Placement in patients with an eGFR
   - Building or enhancing a midline program
   - Building or enhancing peripheral access solutions for patients with difficult venous access (e.g. ultrasound guided IVs, difficult IV access teams/protocols)
   - Other

7. For quality improvement project(s) related to the use of MAGIC or a related decision-tool (e.g. INS Standards) to determine PICC appropriateness, please describe below.

8. Please upload a copy of the decision-tool to determine PICC appropriateness (i.e. high resolution screenshot of decision tool in EMR, hard copy of decision tool given to providers/vascular access/etc., workflow document or other supporting documentation)

9. For quality improvement project(s) related to reducing short term PICC use (PICC <=5 days), please describe below.

10. For quality improvement project(s) related to increasing use of single lumen PICCs (decreasing use of multi-lumen PICCs), please describe below.

11. For quality improvement project(s) related to avoiding PICC placement in patients with eGFR less than 45 ml/min, please describe below.

12. For other quality improvement project(s) related to PICCs, please describe below.

13. Has your hospital encountered any barriers to implementing interventions related to the PICC Tier 1 Interventions?

   - Yes
   - No
   - N/A- Have not implemented an intervention related to the PICC Tier 1 Interventions

14. Please list the barriers encountered related to the PICC Tier 1 Interventions (check all that apply).

   - Leadership commitment
   - Financial obstacles
   - Lack of buy in from vascular access team
   - Lack of buy in from interventional radiology
   - Lack of physician/hospitalist buy in or support
   - Other

15. Please describe the other barriers encountered related to the PICC Tier 1 Interventions. (If “other” selected for barriers encountered related to the PICC Tier 1 interventions.

16. Has your hospital worked on quality improvement project(s) related to any of the following PICC Tier 2 Interventions? (Check all that apply.)

   - Catheter Occlusion
   - PICC-Related Deep Vein Thrombosis (DVT) or Thromboembolism
   - PICC-Related Bloodstream Infection (CLABSI)
   - None of the above

17. For quality improvement project(s) related to avoiding Catheter Occlusions, please describe below. (If “catheter occlusion” is selected in “Has your hospital worked on quality improvement projects related to any of the following PICC Tier II Interventions?”)

18. For quality improvement project(s) related to avoiding PICC-Related Deep Vein Thrombosis (DVT) or Thromboembolism, please describe below. (If “DVT/Thromboembolism” is selected in “Has your hospital worked on quality improvement projects related to any of the following PICC Tier II Interventions?”)

19. For quality improvement project(s) related to avoiding PICC-Related Bloodstream Infection (CLABSI), please describe below. (If “CLABSI” is selected in “Has your hospital worked on quality improvement projects related to any of the following PICC Tier II Interventions?”)

20. Has your hospital encountered any barriers to implementing interventions related to the PICC Tier 2 Interventions?

- Yes
- No
- N/A- Have not implemented an intervention related to the PICC Tier 2 Interventions

21. Please list the barriers encountered related to the PICC Tier 2 Interventions (check all that apply). (If “yes” is selected in “Has your hospital encountered any barriers to implementing interventions related to the PICC Tier 2 Interventions?”)

- Leadership commitment
- Financial obstacles
- Lack of buy in from vascular access team
- Lack of buy in from interventional radiology
- Lack of physician/hospitalist buy in or support
- Other

22. Please describe the other barrier encountered related to the PICC Tier 2 Interventions. (If “other” is selected in “Please list the barriers encountered related to the PICC Tier 2 Interventions”).

23. Does your hospital have a committee that reviews data related to vascular access devices (including PICCs)?

- Yes
- No

24. How often does the multi-disciplinary team meet to review PICC care and quality concerns? (if “yes” is selected in “Does your hospital have a committee that reviews data related to vascular access devices (including PICCs)?”)

- Quarterly
- Monthly
- Bi- Weekly
- None of the above

25. Please list the specialties of the individuals involved in your committee that reviews data related to vascular access devices (including PICCs). (if “yes” is selected in “Does your hospital have a committee that reviews data related to vascular access devices (including PICCs)?”)

- Leadership
- Intensive Care/Critical Care
- Hospitalists
- Hematology/Oncology
- Interventional Radiology
- Vascular Access Nurse/Team Member
- Nephrology
- Emergency Department Physician/Team Member
- Other

26. For the specialty (leadership) involved in your committee please list the title(s) of this individual(s).

27. For the other specialty involved your committee please list the specialty(ies) of this individual(s).

28. Please upload a copy of the minutes from your recent committee meeting that reviews data related to vascular access devices (including PICCs). (if "yes" is selected in "Does your hospital have a committee that reviews data related to vascular access devices (including PICCs)?")

29. Has any HMS-produced PICC data been shared with your institution (i.e. reports distributed at the HMS collaborative wide meetings and/or hospital level reports in the HMS database)?

- Yes
- No

30. Did you (or a member of your hospital) attend the HMS PICC Tier 1 Webinar or review the webinar recording?

- Yes
- No

31. On a scale from 1-10, please rate the HMS PICC Tier 1 Webinar (1= Poor, 10=Great) (if "yes" is selected in "Did you or a member of your hospital attend the HMS PICC Tier 1 webinar or review the webinar recording?").

32. Do you have any additional feedback/suggested improvements for future webinars focusing on the PICC Interventions? (if "yes" is selected in "Did you or a member of your hospital attend the HMS PICC Tier 1 webinar or review the webinar recording?").

- Yes
- No

33. Please detail your feedback/suggested improvements for future webinars focusing on the PICC interventions. (if "yes" is selected in "Do you have any additional feedback/suggested improvements for future webinars focusing on the PICC interventions?").

<u>HMS Quality Improvement Survey - Fall 2017</u>

As a reminder, the HMS Fall 2017 Quality Improvement Survey contains several questions that pertain to the 2017 Michigan Hospital Medicine Safety Consortium Collaborative Quality Initiative Performance Index Scorecard. If you have any questions related to this, please contact Julie Wietzke (jwietzke@med.umich.edu).

Please answer the following questions based on the activity at your hospital over the last six months (unless otherwise specified). The survey is due November 10, 2017.

1. Hospital Name

PICC Specific Information

2. What was the total number of PICCs placed in adult hospitalized patients by all PICC inserters (e.g. interventional radiology, vascular access, etc) at your hospital over the last 12 months? Please use data from the most current 12 months available. Please exclude midlines or catheter exchanges, if possible.

3. Indicate the following:

   - Exchanges included in total PICC number above.
   - Midlines included in total PICC number above.
   - None of the above

4. What was the total number of non-tunneled central venous catheters placed by all certified central venous catheter inserters in medical patients? Please use data from the most current 12 months available. Please exclude dialysis catheters. (If you are unable to obtain this data, please enter -1.)

5. Please tell us the source of the information related to the total number of PICCs. (Select all that apply.)

   - Vascular Access Team
   - Interventional Radiology Team
   - Billing/Financial Records
   - Material Supplies/Central Sterile Supply
   - Other

6. You indicated "Other" as the source of the information related to the total number of PICCs. Please describe the "Other" method.

7. Which of the following quality improvement project(s) related to the PICC Tier 1 Interventions has your hospital been actively working on? (Check all that apply.)

- Creation of a vascular access committee
- Use of MAGIC or a related decision-tool (e.g. INS Standards) to determine PICC appropriateness
- Reducing Short Term PICC Use
- Increasing use of single lumen PICCs; decreasing use of multi-lumen PICCs
- Avoiding PICC Placement in patients with an eGFR < 45 ml/min
- Building or enhancing a midline program
- Building or enhancing peripheral access solutions for patients with difficult venous access (e.g. ultrasound guided IVs, difficult IV access teams/protocols)
- Other

8. For quality improvement project(s) related to the use of MAGIC or a related decision-tool (e.g. INS Standards) to determine PICC appropriateness, please describe below.

9. Please upload a copy of the decision-tool to determine PICC appropriateness (i.e. high resolution screenshot of decision tool in EMR, hard copy of decision tool given to providers/vascular access/etc., workflow document or other supporting documentation)

10. For quality improvement project(s) related to reducing short term PICC use (PICC <=5 days), please describe below.

11. For quality improvement project(s) related to increasing use of single lumen PICCs (decreasing use of multi-lumen PICCs), please describe below.

12. For quality improvement project(s) related to avoiding PICC placement in patients with eGFR < 45ml/min, please describe below.

13. For other quality improvement project(s) related to PICCs, please describe below.

14. Has your hospital encountered any barriers to implementing interventions related to the PICC Tier 1Interventions?

- Yes
- No
- N/A - Have not implemented an intervention related to the PICC Tier 1 Interventions

15. Please list the barriers encountered related to the PICC Tier 1 Interventions (check all that apply). (If "yes" to "Has your hospital encountered any barriers to implementing interventions related to the PICC Tier 1 Interventions?")

- Leadership commitment
- Financial obstacles
- Lack of buy in from vascular access team
- Lack of buy in from interventional radiology
- Lack of physician/hospitalist buy in or support
- Other

16. Please describe the other barriers encountered related to the PICC Tier 1 Interventions. (If “other” to “Please list the barriers encountered related to the PICC Tier 1 Interventions”)

17. Has your hospital launched any quality improvement project(s) related to the any of the following PICC Tier 2 Interventions? (Check all that apply.)

    - Catheter Occlusion
    - PICC-Related Deep Vein Thrombosis (DVT) or Thromboembolism
    - PICC-Related Bloodstream Infection (CLABSI)
    - None of the above

18. For quality improvement project(s) related to avoiding Catheter Occlusions, please describe below. (If “catheter occlusion” to “Has your hospital launched any quality improvement project(s) related to the any of the following PICC Tier 2 Interventions?”)

19. For quality improvement project(s) related to avoiding PICC-Related Deep Vein Thrombosis (DVT) or Thromboembolism, please describe below. (If “DVT/thromboembolism” to “Has your hospital launched any quality improvement project(s) related to the any of the following PICC Tier 2 Interventions?”)

20. For quality improvement project(s) related to avoiding PICC-Related Bloodstream Infection (CLABSI), please describe below. (If “CLABSI” to “Has your hospital launched any quality improvement project(s) related to the any of the following PICC Tier 2 Interventions?”)

21. Has your hospital encountered any barriers to implementing interventions related to the PICC Tier 2 Interventions?

    - Yes
    - No
    - N/A- Have not implemented an intervention related to the PICC Tier 2 Interventions

22. Please list the barriers encountered related to the PICC Tier 2 Interventions (check all that apply). (If “yes” to “Has your hospital encountered any barriers to implementing interventions related to the PICC Tier 2 Interventions?”)

    - Leadership commitment
    - Financial obstacles
    - Lack of buy in from vascular access team
    - Lack of buy in from interventional radiology
    - Lack of physician/hospitalist buy in or support
    - Other

23. Please describe the other barrier encountered related to the PICC Tier 2 Interventions. (If “other” to “Please list the barriers encountered related to the PICC Tier 2 Interventions.”)

24. Does your hospital have a committee that reviews data related to vascular access devices (including PICCs)?

    - Yes
    - No

25. How often does the multi-disciplinary team meet to review PICC care and quality concerns? (If "yes" to "Does your hospital have a committee that reviews data related to vascular access devices (including PICCs)?")

- Quarterly
- Monthly
- Bi- Weekly
- None of the above

26. Please list the specialties of the individuals involved in your committee that reviews data related to vascular access devices (including PICCs). (If "yes" to "Does your hospital have a committee that reviews data related to vascular access devices (including PICCs)?")

- Leadership
- Intensive Care/Critical Care
- Hospitalists
- Hematology/Oncology
- Interventional Radiology
- Vascular Access Nurse/Team Member
- Nephrology
- Emergency Department Physician/Team Member
- Other

27. For the specialty (leadership) involved in your committee please list the title(s) of this individual(s).

28. For the other specialty involved your committee please list the specialty(ies) of this individual(s).

29. Please upload a copy of the minutes from your recent committee meeting that reviews data related to vascular access devices (including PICCs).

30. Has any HMS-produced PICC data been shared within your institution (i.e. reports distributed at the HMS collaborative wide meetings and/or hospital level reports in the HMS database)?

- Yes
- No

<u>HMS Quality Improvement Survey - Spring 2018</u>

As a reminder, the completion of the HMS Spring 2018 Quality Improvement Survey is part of the completeness & accuracy measure on the 2018 Michigan Hospital Medicine Safety Consortium Collaborative Quality Initiative Performance Index Scorecard. If you have any questions related to this, please contact Elizabeth McLaughlin (emcnair@med.umich.edu).

Please answer the following questions based on the activity at your hospital over the last six months (unless otherwise specified). The survey is due June 1, 2018.

1. Hospital Name

PICC Specific Information

2. What was the total number of PICCs placed in adult hospitalized patients by all PICC inserters (e.g. interventional radiology, vascular access, etc.) at your hospital over the last 12 months? Please use data from the most current 12 months available. Please exclude midlines or catheter exchanges, if possible.

3. Indicate the following:

    - Exchanges included in total PICC number above.
    - Midlines included in total PICC number above.
    - None of the above

4. Please tell us the source of the information related to the total number of PICCs. (Select all that apply.)

    - Vascular Access Team
    - Interventional Radiology Team
    - Billing/Financial Records
    - Material Supplies/Central Sterile Supply
    - Other

5. You indicated "Other" as the source of the information related to the total number of PICCs. Please describe the "Other" method.

6. Which of the following quality improvement project(s) related to the PICC Tier 1 Interventions has your hospital been actively working on? (Check all that apply.)

    - Creation of a vascular access committee
    - Use of MAGIC or a related decision-tool (e.g. INS Standards) to determine PICC appropriateness
    - Reducing short term PICC use
    - Increasing use of single lumen PICCs; decreasing use of multi-lumen PICCs
    - Avoiding PICC placement in patients with an eGFR < 45 ml/min
    - Building or enhancing a midline program
    - Building or enhancing peripheral access solutions for patients with difficult venous access (e.g. ultrasound guided IVs, difficult IV access teams/protocols)
    - Other

7. For quality improvement project(s) related to the use of MAGIC or a related decision-tool (e.g. INS Standards) to determine PICC appropriateness, please describe below.

8. For quality improvement project(s) related to reducing short term PICC use (PICC <=5 days), please describe below.

9. For quality improvement project(s) related to increasing use of single lumen PICCs (decreasing use of multi-lumen PICCs), please describe below.

10. For quality improvement project(s) related to avoiding PICC placement in patients with eGFR < 45ml/min, please describe below.

11. For "other" quality improvement project(s) related to PICCs, please describe below.

12. Has your hospital encountered any barriers to implementing interventions related to the PICC Tier 1Interventions?

    - Yes
    - No
    - N/A- Have not implemented an intervention related to the PICC Tier 1 Interventions

13. Please list the barriers encountered related to the PICC Tier 1 Interventions (check all that apply). (If your hospital answered “yes” to “Has your hospital encountered any barriers to implementing interventions related to PICC Tier 1 Interventions?”).

    - Leadership commitment
    - Financial obstacles
    - Lack of buy in from vascular access team
    - Lack of buy in from interventional radiology
    - Lack of physician/hospitalist buy in or support
    - Lack of buy in from nephrologists
    - Delays related to IT
    - Frequent turnover of medical staff
    - Other

14. Please describe the "other" barriers encountered related to the PICC Tier 1 Interventions.

15. Has your hospital launched any quality improvement project(s) related to any of the following PICC Tier 2 Interventions? (Check all that apply.)

    - Catheter Occlusion
    - PICC-Related Deep Vein Thrombosis (DVT) or Thromboembolism
    - PICC-Related Bloodstream Infection (CLABSI)
    - None of the above

16. For quality improvement project(s) related to avoiding Catheter Occlusions, please describe below.

17. For quality improvement project(s) related to avoiding PICC-Related Deep Vein Thrombosis (DVT) or Thromboembolism, please describe below.

18. For quality improvement project(s) related to avoiding PICC-Related Bloodstream Infection (CLABSI), please describe below.

19. Has your hospital encountered any barriers to implementing interventions related to the PICC Tier 2 Interventions?

- Yes
- No
- N/A- Have not implemented an intervention related to the PICC Tier 2 Interventions

20. Please list the barriers encountered related to the PICC Tier 2 Interventions (check all that apply).

- Leadership commitment
- Financial obstacles
- Lack of buy in from vascular access team
- Lack of buy in from interventional radiology
- Lack of physician/hospitalist buy in or support
- Other

21. Please describe the other barriers encountered related to the PICC Tier 2 Interventions.

22. Does your hospital have a committee that reviews data related to vascular access devices (including PICCs)?

- Yes
- No

23. Please list the specialties of the individuals involved in your committee that reviews data related to vascular access devices (including PICCs). (If “yes” to “Does your hospital have a committee that reviews data related to vascular access devices (including PICCs)?”).

- Leadership
- Intensive Care/Critical Care
- Hospitalists
- Hematology/Oncology
- Interventional Radiology
- Vascular Access Nurse/Team Member
- Nephrology
- Emergency Department Physician/Team Member
- Other

24. For the other specialty involved your committee please list the specialty(ies) of this individual(s).

25. Please upload a copy of the minutes from your recent committee meeting that reviews data related to vascular access devices (including PICCs). (If “yes” to “Does your hospital have a committee that reviews data related to vascular access devices (including PICCs)?”).

26. Has your hospital identified a need for any PICC specific tools/interventions that the HMS Coordinating Center can assist with the creation of?

- Yes
- No

27. You indicated a need for PICC specific tools/interventions, please describe.

28. Does your hospital have any PICC specific tools/interventions that you are willing to share with the Collaborative?

- Yes
- No

29. You indicated that your hospital is willing to share PICC specific tools/interventions. Please describe this tool/intervention and a member of the HMS Coordinating Center will be in contact with you.

<u>HMS Quality Improvement Survey - Fall 2018</u>

As a reminder, the completion of the HMS Fall 2018 Quality Improvement Survey is part of the completeness & accuracy measure on the 2018 Michigan Hospital Medicine Safety Consortium Collaborative Quality Initiative Performance Index Scorecard. If you have any questions related to this, please contact Elizabeth McLaughlin (emcnair@med.umich.edu).

Please answer the following questions based on the activity at your hospital over the last six months (unless otherwise specified). The survey is due November 21, 2018.

1. Hospital Name

PICC Specific Information

2. What was the total number of PICCs placed in adult hospitalized patients by all PICC inserters (e.g. interventional radiology, vascular access, etc.) at your hospital over the last 12 months? Please use data from the most current 12 months available. Please exclude midlines or catheter exchanges, if possible.

3. Indicate the following:

   - Exchanges included in total PICC number above.
   - Midlines included in total PICC number above.
   - None of the above

4. Please tell us the source of the information related to the total number of PICCs. (Select all that apply.)

   - Vascular Access Team
   - Interventional Radiology Team
   - Billing/Financial Records
   - Material Supplies/Central Sterile Supply
   - Other

5. You indicated "Other" as the source of the information related to the total number of PICCs. Please describe the "Other" method.

6. Since the beginning of the HMS PICC Initiative, has the total number of PICCs placed at your institution:

   - Increased
   - Decreased
   - Stayed the same

7. Of all CVCs placed in your hospital, what proportion/percentage do you think are PICCs?

   - <20%
   - 20-40%
   - 40-60%
   - 60% or greater

8. Please select the PICC brand/product(s) that are currently utilized (or plan to be utilized) at your hospital?

- BARD- PowerPICC SOLO 2 Catheter
- BARD- PowerPICC Catheter
- BARD- PowerPICC SV Catheter
- BARD- Poly RadPICC Catheter
- BARD- PowerPICC Provena Catheter
- BARD- PowerGroshong PICC Catheter
- BARD- Groshong PICC Catheter
- BARD- Poly Per-Q-Cath PICC Catheter
- BARD- Per-Q- Cath PICC Catheter9
- Cook – Turbo-Ject Power Injectable PICC Catheter
- Cook - Spectrum Turbo-Ject PICC Set – Minocycline/Rifampin Impregnated PICC Catheter
- Cook - Spectrum Silicone Minocycline + Rifampin Impregnated PICC Catheter
- Cook - Silicone Peripherally Inserted Central Venous Catheter
- Cook - Turbo-Flo Peripherally Inserted Central Venous Catheter
- Arrow – Arrow Peripherally Inserted Central Catheter (PICC)
- Arrow – Arrow PICC with Chlorag+ard Technology
- Arrow – Arrow PICC with Chlorag+ard Technology with Pressure Injectable Catheter with BlueFlexTip
- Angiodynamics – BioFlo PICC Catheter
- Navilyst - Xcela PICC with PASV Valve Technology
- Navilyst – Xcela Hybrid PICC with PASV Valve Technology
- Navilyst - Xcela Power Injectable PICC
- Navilyst - Vaxcel PICC
- Navilyst - Vaxcel PICC with PASV Valve Technology
- Other

9. Please list the other PICC brand/product(s) that are currently utilized (or plan to be utilized) at your hospital?

10. Does your hospital use a catheter securement device?

- Yes
- No
- Unknown

11. Please select the type of catheter securement device(s) that are currently utilized (or plan to be utilized) at your hospital? Check all that apply. (if "yes" selected for "Does your hospital use a catheter securement device?")

    - Non-sterile tape (e.g. Micropore)
    - Tegaderm/Transparent dressing/Adhesive-based securement
    - 3M Securement Device
    - Statlock
    - Grip-Lok
    - WingGuard
    - SorbaView SHIELD
    - Secura-cath
    - Steri-strips
    - Suture
    - Other

12. Please list the other catheter securement device(s) that are currently utilized (or plan to be utilized) at your hospital?

13. With regards to de-clotting of catheter occlusions, please select the following answer that is consistent with your hospital's standard of practice.

    - De-clotting is performed only on the occluded lumen
    - De-clotting is performed on all of the lumens for the device that is occluded
    - My hospital does not have a standard of practice related to this
    - Unknown

14. Which of the following quality improvement project(s) related to the PICC Tier 1 Interventions has your hospital been actively working on? (Check all that apply.)

    - Creation of a vascular access committee
    - Use of MAGIC or a related decision-tool (e.g. INS Standards) to determine PICC appropriateness
    - Reducing short term PICC use
    - Increasing use of single lumen PICCs; decreasing use of multi-lumen PICCs
    - Avoiding PICC placement in patients with an eGFR < 45 ml/min
    - Building or enhancing a midline program
    - Building or enhancing peripheral access solutions for patients with difficult venous access (e.g. ultrasound guided IVs, difficult IV access teams/protocols)
    - Other

15. For quality improvement project(s) related to the use of MAGIC or a related decision-tool (e.g. INS Standards) to determine PICC appropriateness, please describe below.

16. For quality improvement project(s) related to reducing short term PICC use (PICC <=5 days), please describe below.

17. For quality improvement project(s) related to increasing use of single lumen PICCs (decreasing use of multi-lumen PICCs), please describe below.

18. For quality improvement project(s) related to avoiding PICC placement in patients with eGFR <45ml/min, please describe below.

19. For "other" quality improvement project(s) related to PICCs, please describe below.

20. Has your hospital encountered any barriers to implementing interventions related to the PICC Tier 1 Interventions?

- Yes
- No
- N/A- Have not implemented an intervention related to the PICC Tier 1 Interventions

21. Please list the barriers encountered related to the PICC Tier 1 Interventions (check all that apply).

- Leadership commitment
- Financial obstacles
- Lack of buy in from vascular access team
- Lack of buy in from interventional radiology
- Lack of physician/hospitalist buy in or support
- Lack of buy in from nephrologists
- Delays related to IT
- Frequent turnover of medical staff
- Other

22. Please describe the "other" barriers encountered related to the PICC Tier 1 Interventions.

23. Has your hospital launched any quality improvement project(s) related to any of the following PICC Tier 2 Interventions? (Check all that apply.)

- Catheter Occlusion
- PICC-Related Deep Vein Thrombosis (DVT) or Thromboembolism
- PICC-Related Bloodstream Infection (CLABSI)
- None of the above

24. For quality improvement project(s) related to avoiding Catheter Occlusions, please describe below. (if “catheter occlusion” was selected from “Has your hospital launched any quality improvement projects related to any of the following PICC Tier 2 Interventions?”)

25. For quality improvement project(s) related to avoiding PICC-Related Deep Vein Thrombosis (DVT) or Thromboembolism, please describe below. (if “DVT/thromboembolism” was selected from “Has your hospital launched any quality improvement projects related to any of the following PICC Tier 2 Interventions?”)

26. For quality improvement project(s) related to avoiding PICC-Related Bloodstream Infection (CLABSI), please describe below. (if “CLABSI” was selected from “Has your hospital launched any quality improvement projects related to any of the following PICC Tier 2 Interventions?”)

27. Has your hospital encountered any barriers to implementing interventions related to the PICC Tier 2 Interventions?

- Yes
- No
- N/A- Have not implemented an intervention related to the PICC Tier 2 Interventions

28. Please list the barriers encountered related to the PICC Tier 2 Interventions (check all that apply). (if “yes” selected for “Has your hospital encountered any barriers to implementing interventions related to the PICC Tier 2 Intervention?”)

- Leadership commitment
- Financial obstacles
- Lack of buy in from vascular access team
- Lack of buy in from interventional radiology
- Lack of physician/hospitalist buy in or support
- Other

29. Please describe the other barriers encountered related to the PICC Tier 2 Interventions.

30. Does your hospital have a committee that reviews data related to vascular access devices (including PICCs)?

- Yes
- No

31. Has your hospital identified a need for any PICC specific tools/interventions that the HMS Coordinating Center can assist with the creation of?

- Yes
- No

32. You indicated a need for PICC specific tools/interventions, please describe. (If “yes” to “Has your hospital identified a need for any PICC specific tools/interventions that the HMS Coordinating Center can assist with the creation of?”)

33. Does your hospital have any PICC specific tools/interventions that you are willing to share with the Collaborative?

- Yes
- No

34. You indicated that your hospital is willing to share PICC specific tools/interventions. Please describe this tool/intervention and a member of the HMS Coordinating Center will be in contact with you.
