# Supplementary material for: Barriers and facilitators for implementing peripherally inserted central catheter (PICC) appropriateness guidelines: A longitudinal survey study from 34 Michigan hospitals
Source: PLoS One. 2022 Nov 4;17(11):e0277302. doi: 10.1371/journal.pone.0277302 (PMC9635738; doi:10.1371/journal.pone.0277302)
Supplement: S1 Table — (DOCX) [file pone.0277302.s002.docx]

**S1 Table. Hospital Demographics**

| **Site ID** | **Bed Size^1^** | **Total Patient Discharges^2^** | **Teaching Hospital Status^3^** | **Ownership Status^4^** |
| --- | --- | --- | --- | --- |
| 1 | 250–499 | 10,000 to 19,999 | Yes | Non-profit |
| 2 | ≥ 500 | ≥ 20,000 | Yes | Non-profit |
| 3 | 250–499 | 10,000 to 19,999 | Yes | For-profit |
| 4 | 250–499 | 10,000 to 19,999 | Yes | Non-profit |
| 5 | 250–499 | 10,000 to 19,999 | Yes | For-profit |
| 6 | 250–499 | < 10,000 | Yes | Non-profit |
| 7 | < 250 | < 10,000 | Yes | Non-profit |
| 8 | ≥ 500 | ≥ 20,000 | Yes | Non-profit |
| 9 | 250–499 | 10,000 to 19,999 | Yes | Non-profit |
| 10 | ≥ 500 | ≥ 20,000 | Yes | Non-profit |
| 11 | < 250 | < 10,000 | No | Non-profit |
| 12 | < 250 | 10,000 to 19,999 | Yes | Non-profit |
| 13 | < 250 | < 10,000 | No | Non-profit |
| 14 | < 250 | < 10,000 | Yes | Non-profit |
| 15 | ≥ 500 | ≥ 20,000 | Yes | Non-profit |
| 16 | 250–499 | ≥ 20,000 | No | Non-profit |
| 17 | 250–499 | ≥ 20,000 | No | Non-profit |
| 18 | < 250 | < 10,000 | Yes | For-profit |
| 19 | ≥ 500 | ≥ 20,000 | Yes | Non-profit |
| 20 | < 250 | < 10,000 | Yes | Non-profit |
| 21 | 250–499 | 10,000 to 19,999 | Yes | Non-profit |
| 22 | < 250 | < 10,000 | No | Non-profit |
| 23 | ≥ 500 | ≥ 20,000 | Yes | Non-profit |
| 24 | 250–499 | 10,000 to 19,999 | Yes | Non-profit |
| 25 | ≥ 500 | 10,000 to 19,999 | Yes | For-profit |
| 26 | ≥ 500 | ≥ 20,000 | Yes | Non-profit |
| 27 | 250–499 | 10,000 to 19,999 | Yes | Non-profit |
| 28 | 250–499 | 10,000 to 19,999 | Yes | Non-profit |
| 29 | < 250 | < 10,000 | No | Non-profit |
| 30 | < 250 | 10,000 to 19,999 | No | Non-profit |
| 31 | < 250 | < 10,000 | No | Non-profit |
| 32 | < 250 | < 10,000 | Yes | Non-profit |
| 33 | ≥ 500 | ≥ 20,000 | Yes | Non-profit |
| 34 | 250–499 | 10,000 to 19,999 | Yes | Non-profit |

^1^Data obtained from 2019 Michigan Certificate of Need Annual Survey, Basic Total Licensed Beds Utilization Statistics. Retrieved 4/21/2021 from <https://www.michigan.gov/documents/mdhhs/Report_010_Hospital_Beds_by_HSA_703357_7.pdf>

*^2^*Data obtained from American Hospital Directory, Inc.’s *Individual Hospital Statistics for Michigan.* Retrieved 4/22/2021 from <https://www.ahd.com/states/hospital_MI.html>

^3^Data obtained from AMA’s Data Hub. Retrieved 4/28/2021 from <https://guide.prod.iam.aha.org/guide/searchResults>

^4^Data obtained from AMA’s Data Hub. Retrieved 4/28/2021 from <https://guide.prod.iam.aha.org/guide/searchResults>
